# Direct communication between osteocytes and acid-etched titanium implants with a sub-micron topography

**DOI:** 10.1007/s10856-016-5779-1

**Published:** 2016-10-03

**Authors:** Furqan A. Shah, Patrik Stenlund, Anna Martinelli, Peter Thomsen, Anders Palmquist

**Affiliations:** 1 Department of Biomaterials, Sahlgrenska Academy at University of Gothenburg, Göteborg, Sweden; 2 BIOMATCELL VINN Excellence Centre of Biomaterials and Cell Therapy, Göteborg, Sweden; 3 Department of Chemistry, Materials and Surfaces, SP Technical Research Institute of Sweden, Borås, Sweden; 4 Department of Chemistry and Chemical Engineering, Chalmers University of Technology, Göteborg, Sweden

## Abstract

The osteocyte network, through the numerous dendritic processes of osteocytes, is responsible for sensing mechanical loading and orchestrates adaptive bone remodelling by communicating with both the osteoclasts and the osteoblasts. The osteocyte network in the vicinity of implant surfaces provides insight into the bone healing process around metallic implants. Here, we investigate whether osteocytes are able to make an intimate contact with topologically modified, but micrometre smooth (*S*_a_ < 0.5 µm) implant surfaces, and if sub-micron topography alters the composition of the interfacial tissue. Screw shaped, commercially pure (cp-Ti) titanium implants with (i) machined (*S*_a_ = ~0.2 µm), and (ii) two-step acid-etched (HF/HNO_3_ and H_2_SO_4_/HCl; *S*_a_ = ~0.5 µm) surfaces were inserted in Sprague Dawley rat tibia and followed for 28 days. Both surfaces showed similar bone area, while the bone-implant contact was 73 % higher for the acid-etched surface. By resin cast etching, osteocytes were observed to maintain a direct intimate contact with the acid-etched surface. Although well mineralised, the interfacial tissue showed lower Ca/P and apatite-to-collagen ratios at the acid-etched surface, while mineral crystallinity and the carbonate-to-phosphate ratios were comparable for both implant surfaces. The interfacial tissue composition may therefore vary with changes in implant surface topography, independently of the amount of bone formed. Implant surfaces that influence bone to have higher amounts of organic matrix without affecting the crystallinity or the carbonate content of the mineral phase presumably result in a more resilient interfacial tissue, better able to resist crack development during functional loading than densely mineralised bone.

## Introduction

The osteocyte network is responsible for sensing mechanical loading and orchestrates adaptive bone remodelling by communicating with both the osteoclasts and the osteoblasts [1]. Osteocyte processes (also known as dendrites) reside within interconnecting channels called canaliculi, and are frequently extended and retracted [2]. Although osteocytes may sense mechanical loading in several ways [3], the dendritic processes are indicated to be of considerable importance [4]. Furthermore, osteocytes in the vicinity of implant surfaces provide insight into the bone healing process around metallic implants [5].

Topographical features such as undercuts on the sub-micron scale present a three dimensional structure with which the extracellular matrix of newly formed bone can establish mechanical interlocking [6], thus strongly influencing the bone-bonding ability of implant surfaces. Subtractive surface modification (i.e., acid-etching) has a positive effect on the strength of endosseous integration [7]. And indeed, modification of implant surface topography is frequently carried out by the use of acids such as HCl, H_2_SO_4_, HNO_3,_ and HF [8].

At early healing times, topologically optimised surfaces are commonly said to induce advantageous biological responses, e.g., rapid bone formation. Histological differences between topologically modified and unmodified (machined) surfaces are typically less pronounced at late healing times. Moreover, histological methods employing optical microscopy are less sensitive to subtle localised variations at smaller length scales (i.e., micron-, sub-micron, and nano-), e.g., the extracellular matrix composition and ultrastructure. If specific surface modifications are able to continually induce a desirable biological response throughout the effective lifetime of an implanted device, e.g., higher bone remodelling and/or bone turnover, beyond merely faster initial bone formation, this may be reflected in the composition and ultrastructure of not only the interfacial tissue, but also up to several tens of micrometres from the implant surface.

With acid-etching being a frequently employed method to intentionally roughen implant surfaces, little is known about the composition of the bone-implant interface in relation to such surfaces [9]. Roughened surfaces have been shown to affect both the stiffness and the hardness of bone [10], and the biomechanical anchorage of the implant in bone as determined from removal torque measurements [7]. Indeed, mechanical interlocking between the implant surface and bone contributes towards the observed increase in the force required to disrupt the interface and unscrew the implant [11]. A key question therefore is whether sub-micron topography also modulates the molecular composition of the interfacial tissue. Furthermore, are osteocytes able to establish and maintain an intimate contact with topologically modified, but micrometre smooth (*S*_a_ < 0.5 µm [12]) implant surfaces?

## Materials and methods

### Implant fabrication and characterisation of surface morphology

Thirty-six screw-shaped implants were machined from a commercially pure (cp-Ti) titanium rod (Elos Medtech Pinol A/S, Gørløse, Denmark). Half of the implants were dual acid-etched in two steps, using HF/HNO_3_ followed by H_2_SO_4_/HCl. The remaining implants were left as machined. All implants were ultrasonically cleaned in Milli-Q water, organic solvents, and steam sterilised. Two implants of each type were used for surface characterisation.

Qualitative visualisation of the implant surface morphology was performed using scanning electron microscopy (SEM; Zeiss SUPRA^®^ 40 VP, Germany) operated in the secondary electron mode at 5 kV acceleration voltage. For quantitative assessment of surface topography, several parameters were investigated at the top, flank, and valley regions of the implant threads, on two implants of each type by 3D-SEM. For 3D reconstruction, images were acquired with an 8° eucentric tilt and processed by software (MeX^®^ 6.0, Alicona Imaging GmbH, Graz).

### Animal surgery and sample preparation

The implants were placed in the proximal and distal tibial metaphyses of eight skeletally-mature Sprague Dawley rats (two implants in each tibia) and were followed for four weeks. The experiment was approved by the local Animal Ethics Committee at the University of Gothenburg (Dnr 279-2011). Prior to surgery, the animals were anaesthetised by isoflurane inhalation (Isoba^®^ Vet; Schering-Plough, Uxbridge, UK) and were administered buprenorphine hydrochloride (Temgesic, 0.03 mg/kg; Reckitt & Colman, Hull, UK), subcutaneously, directly postoperatively and for the following two days. The animals were fed ad libitum. The animals were euthanised with an intraperitoneal overdose of sodium pentobarbital (60 mg/mL; ATL Apoteket Production & Laboratories, Sweden). The fixative solution (~200 mL) was perfused via the heart. After removing the skin and the surrounding soft tissue, the implants were retrieved with surrounding bone, immersion fixed for one week, dehydrated in a graded series of ethanol and resin embedded [13].

### Qualitative histology

Central ground sections were prepared from selected sample pairs (*n* = 4) and stained with toluidine blue for qualitative histology using optical microscopy (Nikon Eclipse E600; Nikon NIS-Elements software).

### Electron microscopy

Other resin embedded implants were bisected longitudinally by sawing and polished with 400–2400 grit SiC paper. Backscattered electron scanning electron microscopy (BSE-SEM, FEI Quanta 200 FEG ESEM) was performed at low vacuum, 20 kV accelerating voltage and 10 mm working distance. Images obtained at ×500 magnification were used for quantitative histomorphometry (*n* = 9) to measure the bone area (BA) and the bone-implant contact (BIC), by semi-automated segmentation using Adobe Photoshop CS 5.1, ImageJ (imagej.nih.gov/ij), and the Image Edge plugin for edge detection.

Energy dispersive X-ray spectroscopy (INCA 300 EDX system, Oxford Instruments GmbH, Wiesbaden, Germany) was performed at 0–10 keV spectral range to determine the Ti, Ca, P, and O content of the interfacial tissue at each implant type (*n* = 2). At high magnification, a 5 × 5 point grid-matrix (enclosing ~1 µm^2^) with equal vertical and horizontal spacing was placed in an osteocyte-free zone ≤1 μm away from the tissue edge. Owing to the presence of a separation artefact between the tissue and the implant surface, the analysis was carried out only in areas that had separated by ≤2 μm. For each implant (of either type), five locations along the implant thread were analysed (i.e., a total of ten locations per group). As a reference (and not considered for statistical analysis), two locations in the native bone were also analysed for each implant.

An electron transparent specimen (150–200 nm thick) was prepared across the bone-implant interface using a focused ion beam in situ lift-out technique (Strata DB235 FIB/SEM; FEI Company, The Netherlands) [14]. Scanning transmission electron microscopy (STEM) was performed in the high-angle annular dark field (HAADF) mode (Tecnai T20, FEI Company, The Netherlands) to visualise the adaptation of bone to the sub-micron topography. Elemental analysis of the newly formed bone was performed using energy dispersive X-ray spectroscopy (STEM-EDX).

### Direct visualisation of osteocyte morphology

Direct visualisation of osteocytes within the newly formed bone tissue was enabled after resin cast etching [5]. Briefly, polished bone-implant blocks were sequentially immersed in 9 % H_3_PO_4_ and 5 % NaOCl, air-dried overnight and Au sputter-coated (~10 nm) for high vacuum secondary electron SEM (Ultra 55 FEG SEM, Leo Electron Microscopy Ltd, UK).

### Raman spectroscopy

For non-destructive investigation of the mineralisation process at the bone-implant interface [15], Raman spectroscopy (InVia Reflex Raman spectrometer, Renishaw, UK) was performed on polished bone-implant blocks using a 785 nm excitation laser, an 1800 1/mm grating, and averaging five scans of 20 s at each location. The nominal spectral resolution at these conditions is close to 1 cm^−1^. Raman spectra were processed using the Background Correction program [16] for MATLAB R2013b (Mathworks Inc., Natick, MA). The baseline-subtracted spectra were normalised to the intensity of the ν_1_ PO_4_^3−^ band at ~960 cm^−1^ using Plot (http://plot.micw.eu/). The major peak assignments were 432 cm^−1^ (ν_2_ PO_4_^3−^), 579 cm^−1^ (ν_4_ PO_4_^3-−^), 960 cm^−1^ (ν_1_ PO_4_^3−^), 1070 cm^−1^ (ν_1_ CO_3_^2−^), 855 cm^−1^ (proline), 875 cm^−1^ (hydroxyproline), 1004 cm^−1^ (phenylalanine), 1240–1270 cm^−1^ (Amide III), 1447 cm^−1^ (methylene *δ*(CH_2_) scissoring), and 1650 cm^−1^ (Amide I). The mineral crystallinity, taken as the inverse full-width at half-maximum of the ν_1_ PO_4_^3−^ band (1/FWHM) [17], the apatite-to-collagen (ν_2_ PO_4_^3−^/Amide III) ratio [18], and the carbonate-to-phosphate (ν_1_ CO_3_^2−^/ν_1_ PO_4_^3−^) ratio [19] were also quantified.

### Statistical analysis

The Kruskal-Wallis test followed by the Mann-Whitney U test were used for all statistical analyses between the implant types for quantitative histomorphometry, EDX, and the Raman metrics (SPSS Statistics, v.23, IBM Corporation); *P* values < 0.05 were considered statistically significant. Mean values ± standard deviations are presented.

## Results

### Surface topography characterisation

**Fig 1:**
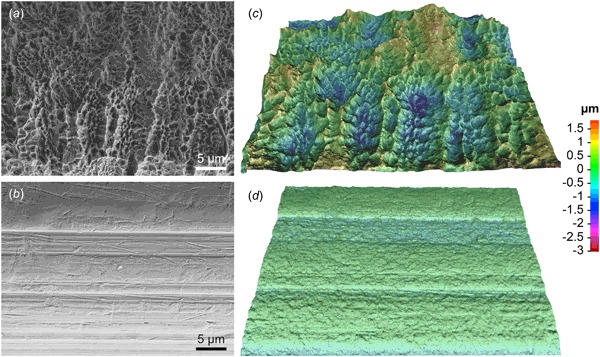
Secondary electron SEM images of the **a** acid-etched and the **b** machined implant surfaces. 3D-SEM reconstruction of the **c** acid-etched and the **d** machined implant surfaces. The *colour*-coding represents the surface topography superimposed onto the macro-shape of the implant thread

The acid-etched surface exhibited morphological changes attributable to the etching process (Fig. 1a, b), where sub-micron (200–500 nm diameter) pits with sharp walls in-between dominated the implant surface. The machined surface mainly displayed scratches and ridges along the machining direction with a relatively featureless anisotropic topography. An underlying waviness was observed perpendicular to the machining direction, most likely originating from the microstructure with elongated grains in the wire drawing direction. Quantitative 3D-SEM confirmed the higher roughness of the acid-etched surface compared to the machined surface (Table 1). Based on the *S*_a_ values, both implant surfaces are considered *smooth* in accordance with Albrektsson and Wennerberg [12].

**Table 1:** Surface topography characterisation (mean values ± SD)

Parameter	S_a_ (nm)	S_p_ (µm)	S_v_ (µm)	S_10z_ (µm)	S_dq_	S_dr_ (%)
Acid-etched	456 ± 80	1.79 ± 0.14	1.66 ± 0.33	3.21 ± 0.34	0.91 ± 0.06	36.09 ± 4.52
Machined	199 ± 122	0.83 ± 0.40	0.54 ± 0.12	1.33 ± 0.50	0.46 ± 0.09	9.65 ± 3.85

Geometric means of values obtained from two separate implants. S_a_: Arithmetic mean deviation of the surface, S_p_: Maximum peak height of the surface, S_v_: Maximum valley depth of the surface, S_10z_: Ten point height of the surface, S_dq_: Root-mean-square slope of the surface, S_dr_: Developed surface area ratio

### Histology and histomorphometry

**Fig 2:**
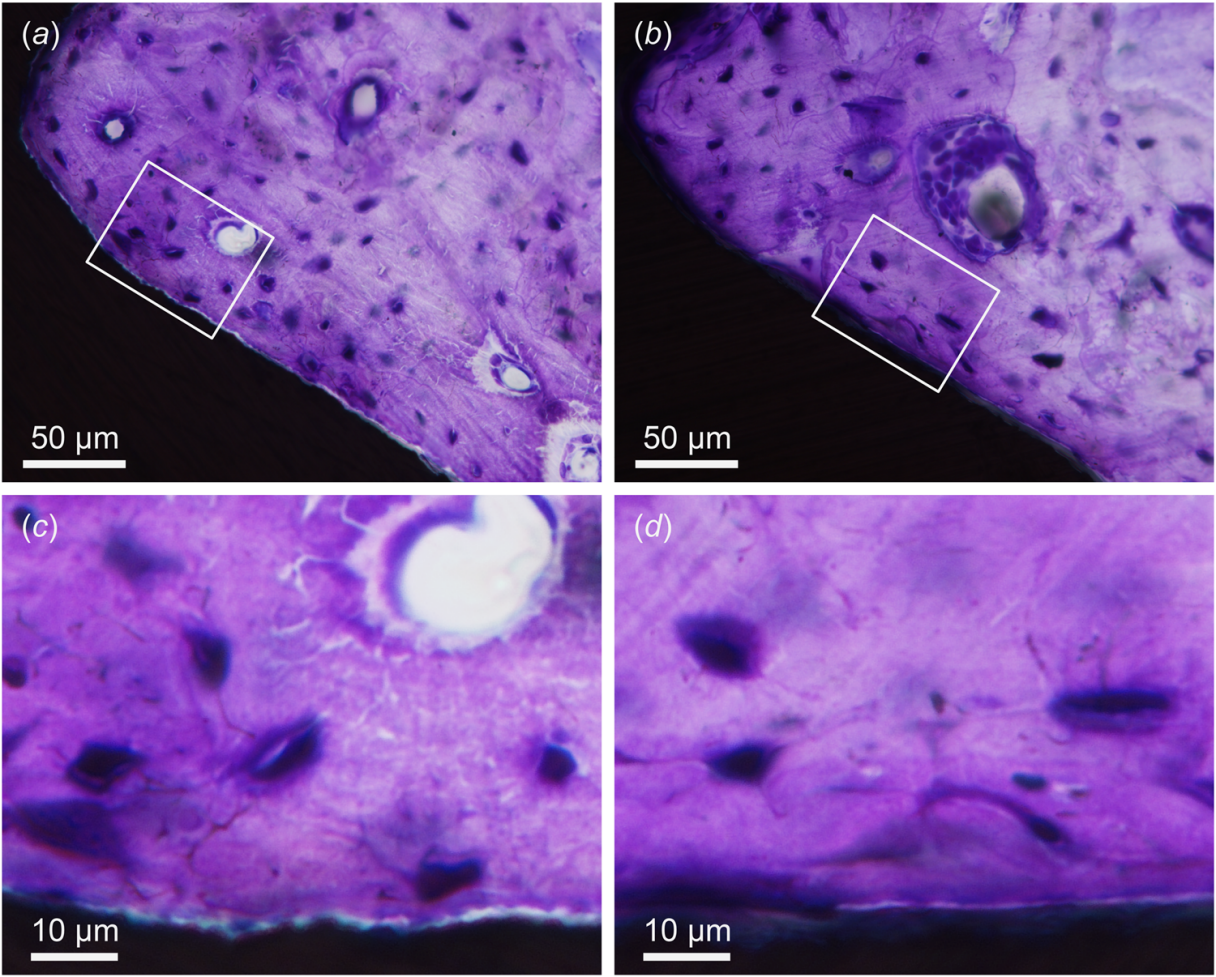
Qualitative histology. High amounts of remodelled, lamellar bone within the cortical threads of the **a** acid-etched and the **b** machined implant surfaces. Osteocytes are found in close vicinity to the **c** acid-etched and the **d** machined implant surfaces

For both implant surfaces, threads located in cortical bone were generally completely filled with high amounts of remodelled, lamellar bone at four weeks of healing (Fig. 2). The threads located in the marrow space, however, showed an endosteal downgrowth of bone along the implant surface. Osteocytes were seen in close proximity to the implant surface. Using BSE-SEM, the acid-etched (38.7 ± 10 %) and the machined (45.2 ± 5.3 %) surfaces showed similar bone area (*P* < 0.15). On the other hand, the bone-implant contact for the acid-etched (83.4 ± 5.1 %) surface was significantly higher (*P* < 0.001) than the machined (48.3 ± 13.5 %) surface.

### Direct visualisation of osteocytes

**Fig 3:**
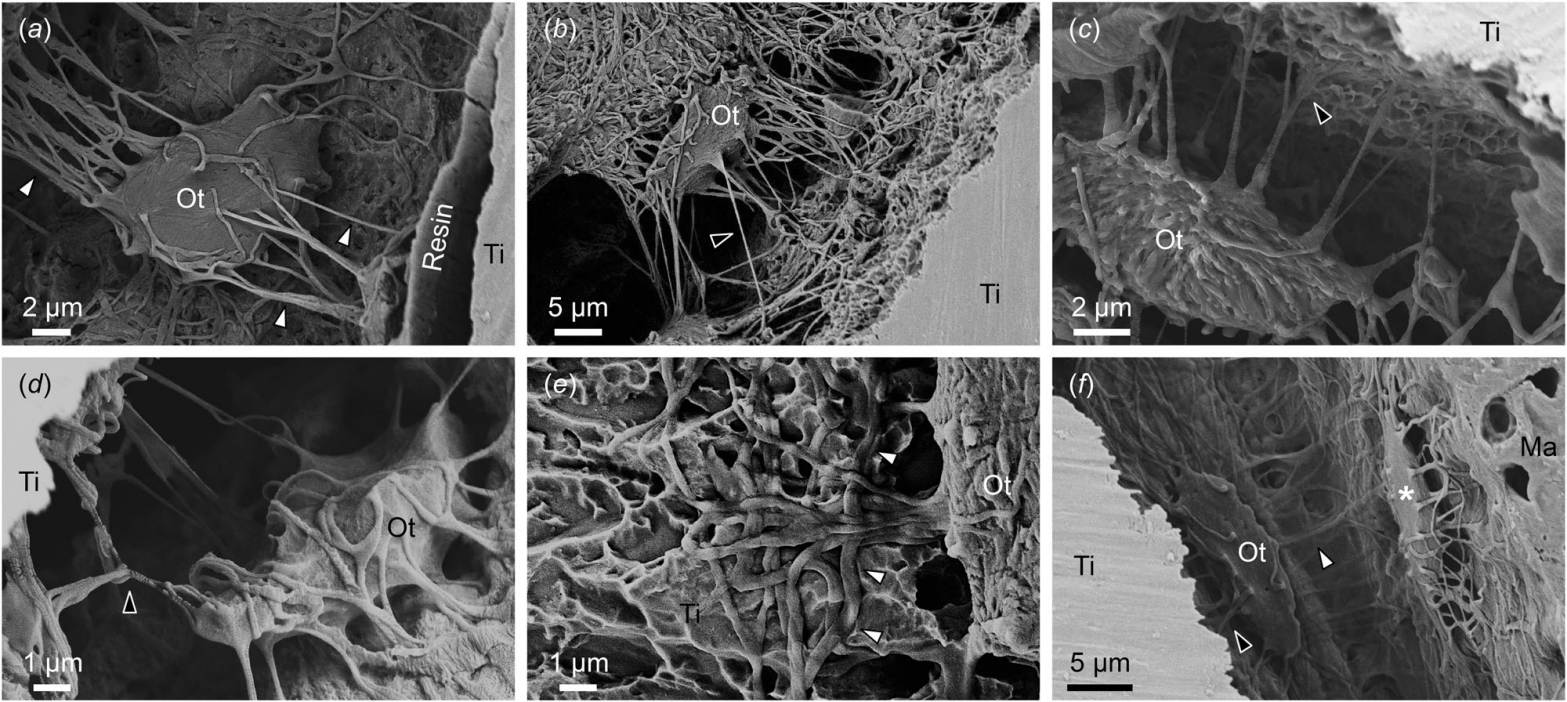
Direct visualisation of osteocytes in close proximity to the implant surface. **a** Poor mechanical interlocking between the machined implant surface and bone results in the formation of a gap (separation artefact) during sample processing. This gap is subsequently occupied by the embedding resin, which appears as a thin film at the bone-implant interface. An osteocyte (Ot) is seen close to the implant surface with several canaliculi extending towards and away from the implant surface (*white arrowheads*). In contrast, no gap (separation artefact) appears adjacent to the acid-etched surface (i.e., a surface that affords better mechanical interlocking) (**b**–**f**). **b** An osteocyte lies with its long axis parallel to the implant surface. Several canaliculi extend towards the implant surface, some stretching up to 20 µm (*black arrowhead*). **c**, **d** Several canaliculi extend towards the acid-etched implant surface, branching (*black arrowheads*) and making an intimate contact to the sub-micron texture. **e** Seen from above, an osteocyte rests directly on the acid-etched implant surface and numerous canaliculi are closely adapted (*white arrowheads*) to the topographical features. **f** Osteocyte alignment provides insight into the origins of lamellar structure of bone adjacent to the implant surface. An osteocyte extends numerous canaliculi towards the implant surface while several canaliculi also extend perpendicularly away from the implant surface. Another, presumably younger, osteocyte (*asterisk*) lines the bone marrow (Ma) and is aligned parallel to the lamellar direction

Resin cast etching exposed a single layer of osteocytes below the bone surface. Osteocytes in close proximity to the implant surface extended several canaliculi towards and away from the implant surface (Fig. 3a). A gap (separation artefact) was observed between the machined implant surface and bone, presumably due to tissue shrinkage during sample preparation [20]. This gap is subsequently occupied by the embedding resin [21, 22], which appears as a thin film between the mineralised tissue and the implant surface (Fig. 3a). However, no such gap had formed adjacent to the acid-etched surface.

Osteocytes closest to the implant surface were generally aligned with their long axes parallel to the surface (Fig. 3a–d, f). Numerous canaliculi extended up to 20 µm towards the implant surface (Fig. 3b), some even branching (Fig. 3c, d), and directly contacting the sub-micron textured surface. Seen from above, osteocytes were observed resting directly on the implant surface and numerous canaliculi were closely adapted (white arrowheads) to the acid-etched topographical features (Fig. 3e).

### Electron microscopy

**Fig 4:**
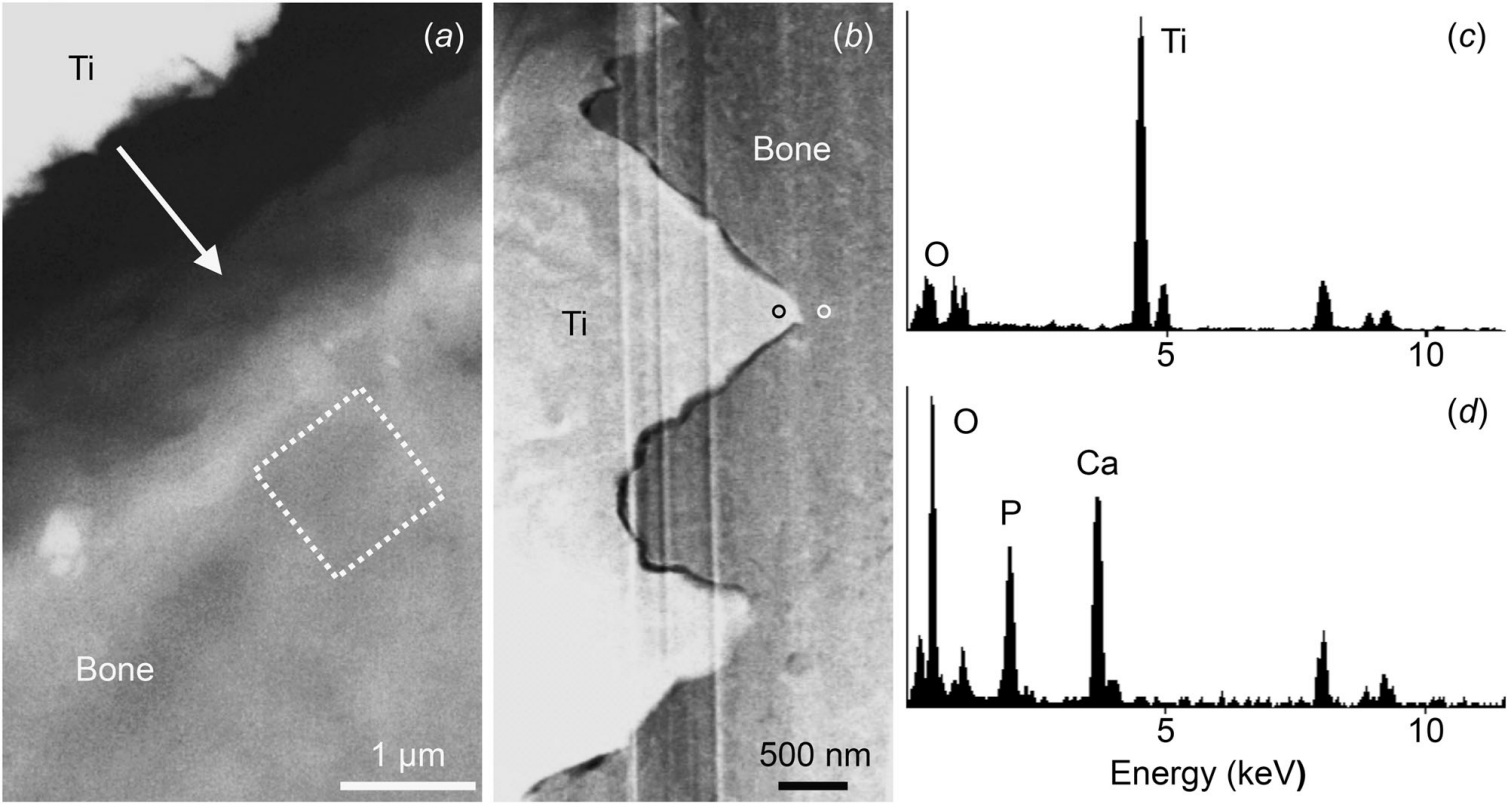
**a** Backscattered electron (BSE-SEM) image showing an osteocyte-free zone along the implant thread where a 5 × 5 point grid-matrix (equal vertical and horizontal spacing) enclosing ~1 µm^2^ was placed (*white box*) for EDX analysis. The *arrow* indicates separation at the bone-implant interface attributable to sample processing procedures. **b** HAADF-STEM image showing the interface tissue well adapted to the sub-micron topography of the acid-etched surface. STEM-EDX analysis of the implant **c** showing high levels of titanium, and oxygen (*black circle* in **b**), and the interfacial tissue **d** adjacent to the implant surface (*white circle* in **b**) confirming the presence of mineralised bone

Considering only the elements Ti, Ca, P, and O, high amounts of Ti were found in the interfacial tissue (Fig. 4a) at both the acid-etched (9.24 ± 1.85 at.%) and the machined (9.41 ± 1.07 at.%) implants. However, Ti was also detected in the native bone outside the acid-etched (1.52 ± 0.16 at.%) and the machined (1.79 ± 0.44 at.%) implant threads. The Ca/P ratio of the interfacial tissue adjacent to the acid-etched surface (1.28 ± 0.11) was significantly lower (*P* = 0.003) than the machined surface (1.47 ± 0.17). The Ca/P ratio of the native bone was 1.54 ± 0.10 (data pooled for both implant types).

In the HAADF-STEM mode, the newly formed bone appeared well adapted to the sub-micron contour of the acid-etched implant surface (Fig. 4b), allowing mechanical interlocking [11]. STEM-EDX analysis indicated high Ca and P content of bone (Fig. 4c, d). No Ti was detected at a distance of 100–150 nm from the implant surface.

### Raman spectroscopy

**Fig 5:**
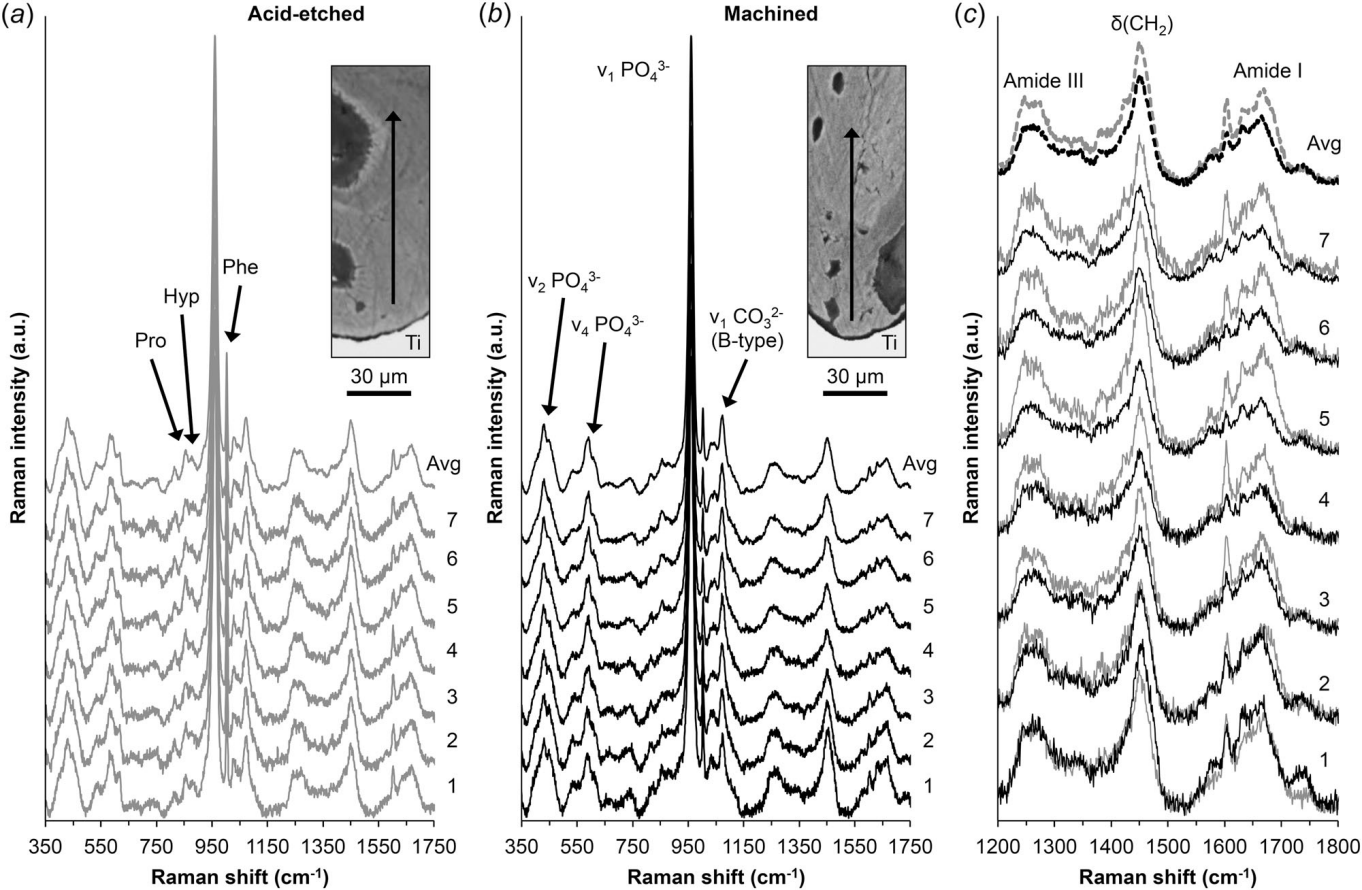
Raman spectra recorded at 10, 25, 40, 55, 70, 85 and 100 µm (indicated as 1–7) from the acid-etched **a** and machined **b** surfaces at the deepest part of the implant threads. All spectra are normalised to the intensity of the ν_1_ PO_4_^3−^ band at ~960 cm^−1^. An averaged spectrum (Avg) of the seven individual spectra is also shown. While mineral crystallinity and the carbonate-to-phosphate ratios were comparable for both implant surfaces, the apatite-to-collagen ratio was marginally higher for the machined implant surface. **c** Detail of the 1200–1800 cm^−1^ region (overlaid spectra: *grey* acid-etched; *black* machined), showing subtle variations in the collagen content

Raman spectra were recorded at 10, 25, 40, 55, 70, 85 and 100 µm (1–7, respectively) from the implant surface in mineralised tissue encompassing two successive lamellar *packets*, avoiding visible osteocyte lacunae, cracks and other unmineralised structures (Fig. 5). The averaged Raman spectra indicated differences in the composition of bone formed up to 100 µm from the two implant surfaces. The apatite-to-collagen ratio was higher (*P* = 0.009) for the machined surface (1.57 ± 0.2) than the acid-etched surface (1.23 ± 0.1). The mineral crystallinity for the machined and the acid-etched surfaces was comparable (FWHM ν_1_ PO_4_^3−^ = 20.48 ± 0.70 and 21.08 ± 0.34, respectively. *P* = 0.085). The carbonate-to-phosphate ratios were also similar (*P* = 0.141) for the machined (0.170 ± 0.004) and the acid-etched (0.166 ± 0.006) surfaces.

Amide bands represent peptide-linkages within proteins and indicate that the helical conformation of type-I collagen molecules remains intact. For the acid-etched surface, the Amide III bands appeared to resolve into two peaks at approximately 1240 and 1270 cm^−1^, which are related to C–N stretching and N–H in-plane deformation modes, respectively [23]. Corresponding to a variation in the BSE *Z*- (atomic number) contrast of the two lamellar *packets* analysed, the difference in the *δ*(CH_2_) and Amide (I and III) signal intensity at 10–40 µm vs. 55–100 µm from the machined surface was quite pronounced (Fig. 5c). In comparison, at 70–100 µm from the acid-etched surface, the Amide III band clearly shows the ν(C–N) and *δ*(N–H) doublet at ~1245 and ~1268 cm^−1^, respectively. The Amide I band shows a shoulder at ~1640 cm^−1^ and an intense peak at ~1664 cm^−1^, assigned to ν(C=C) and ν(C=O) stretching vibrations, respectively.

Raman signatures attributable to amino acids proline (Pro), hydroxyproline (Hyp), and phenylalanine (Phe) were stronger in the bone formed next to the acid-etched surface. These amino acids are the major constituents of type-I collagen and therefore represent the organic matrix. A sharp peak at 1600–1604 cm^−1^ may be assigned to the ring vibration modes of either tyrosine (Tyr) or phenylalanine residues [24]. The intensity of this peak (1600–1604 cm^−1^) seemingly changes proportionately with the 1004 cm^−1^ phenylalanine peak, both of which were higher for the acid-etched surface. The *δ*(CH_2_) peak was also more intense for the acid-etched surface.

## Discussion

After four weeks of healing, the bone-implant contact was significantly higher for the acid-etched surface (vs. the machined surface). At this time point, earlier studies have demonstrated high amounts of bone in direct contact with the implant surface, biomechanical stability of the bone-implant interface, and steady-state bone remodelling based on the relative expression of the genes coding for receptor activator of nuclear factor kappa-B ligand (RANKL) and osteoprotegerin (OPG) [25, 26].

The appearance of a separation artefact during the sample processing steps of fixation and dehydration [20] suggests poor mechanical interlocking between the machined implant surface and bone. On the other hand, the absence of such a gap adjacent to the acid-etched surface is in analogy to previous observations of implant surfaces that yield superior mechanical interlocking [22, 27]. Sample processing procedures (e.g., grinding and polishing) also affect the elemental analysis by SEM-EDX, where titanium could be detected in the native bone, up to several tens of micrometres from the implant surface. In contrast, STEM-EDX did not reveal the presence of titanium within the interfacial tissue (100–150 nm from the implant surface), thereby substantiating the assumption that titanium is smeared across the bone-implant block during the grinding and polishing procedures.

Osteocyte alignment provides valuable insight into the origins of the lamellar structure of bone [28]. During the transition from a predominantly marrow-like tissue towards organised lamellar bone, osteocytes closest to the implant surface originate from the osteoblasts that were the earliest to be recruited towards the surface. It may be speculated that osteoblasts (or precursor cells) attach with their long axes parallel to the implant surface and produce extracellular matrix. Later, a second layer of osteoblasts arrives at the bone formation front while the non-mineralised tissue gradually recedes. Each successive layer/sheet of osteoblasts retains a directional relationship to the underlying bone surface [29]. It is believed that finger-like cytoplasmic extensions beneath the osteoblasts (i.e., on the bone face) form a meshwork of flat processes that assemble collagen fibrils into compact, regularly polarised bundles through temporal and spatial synchronism of groups of osteoblasts. A second set of thinner processes is directed perpendicularly into the depth of the fibrillar matrix, and these processes reside within canaliculi after the osteoblast-osteocyte transformation [30].

The ν_2_ PO_4_^3−^/Amide III ratio is less susceptible to variation with orientation, and therefore provides more accurate information about the composition of bone [31]. In the present work, we observed differences in the apatite-to-collagen ratios for bone formed up to 100 µm from the implant surface. However, the CO_3_^2−^ content remained similar. The carbonate-to-phosphate ratio is a measure of carbonate substitution into the apatite lattice. CO_3_^2−^ predominantly replaces PO_4_^3−^ in biological apatite, known as B-type carbonate substitution [32], thereby affecting various physical properties of apatite, e.g., shortening of the *a*-axis, elongation of the *c*-axis, decreases in crystallite size, thermal stability, solubility etc. [33–35]. These observations suggest that only the mineral fraction varied between the two types of implant surfaces, but the bone apatite itself was similar in terms of mineral crystallinity and the degree of carbonation. While the implant surface may facilitate bone remodelling and maturation at different rates, an alternative explanation may be local variations in the structure and composition of individual *packets* of lamellar bone, as also observed by quantitative backscattered electron imaging (qBEI) [36].

It may be speculated that implant surfaces that influence bone to have higher amounts of organic matrix without affecting the properties of the mineral phase itself (i.e., crystallinity and/or carbonate content) allow the interfacial tissue to be more resilient. Such interfacial tissue may therefore be better suited for load bearing and load transfer from a metal implant into the surrounding bone than densely mineralised tissue which may be brittle and susceptible to crack initiation and propagation.

## Conclusions

After four weeks of healing, similar amounts of new bone had formed at both implant types, while the acid-etched surface showed a 73 % increase in the bone-implant contact. Osteocytes are able to maintain a direct intimate contact with topologically modified, but nominally smooth titanium surfaces prepared by acid-etching. The elemental (e.g., the Ca/P ratio) and molecular composition (e.g., the apatite-to-collagen ratio) of the interfacial tissue may vary with changes in implant surface topography, independently of the amount of bone formed.
